# Endovascular Stenting for Pulmonary Vein Stenosis Following Atrial Fibrillation Ablation: From Diagnosis to Intervention

**DOI:** 10.1002/ccd.70417

**Published:** 2025-12-16

**Authors:** Cristina Aurigemma, Marco Busco, Francesco Bianchini, Gabriella Locorotondo, Francesca Graziani, Annalisa Pasquini, Enrico Romagnoli, Mattia Lunardi, Lazzaro Paraggio, Francesco Varone, Andrea Smargiassi, Bruno Iovene, Riccardo Marano, Carlo Trani, Francesco Burzotta

**Affiliations:** ^1^ Department of Cardiovascular Science Cuore Fondazione Policlinico Universitario A. Gemelli IRCCS Rome Rome Italy; ^2^ Department of Cardiovascular and Pulmonary Sciences Catholic University of the Sacred Heart Rome Italy; ^3^ Department of Neuroscience Sense Organs and Thorax Fondazione Policlinico Universitario A. Gemelli IRCCS Rome Rome Italy; ^4^ Department of Diagnostic Imaging and Oncological Radiotherapy Fondazione Policlinico Universitario A. Gemelli IRCCS Rome Rome Italy

**Keywords:** atrial fibrillation ablation, balloon angioplasty, pulmonary vein occlusion, pulmonary vein stenosis

## Abstract

**Background:**

Pulmonary vein stenosis (PVS) is an uncommon but serious complication of atrial fibrillation (AF) ablation, often misinterpreted as primary pulmonary disease. Timely identification is essential to prevent irreversible injury and to guide appropriate referral for interventional management.

**Case Presentations:**

Through the discussion of two practical case examples, we illustrate the challenges of early recognition, the role of imaging modalities, and the decision‐making process in selecting the most effective interventional strategy. Both young male patients developed progressive dyspnoea, chronic cough, and recurrent pneumonia months after AF ablation and were initially managed for presumed pulmonary pathology. Multimodal imaging—including CT, transoesophageal echocardiography, and selective angiography—confirmed severe multivessel PVS, with a true bifurcation lesion in one case.

**Management Strategy:**

The patients were referred for transcatheter pulmonary angioplasty. Procedures were performed under combined fluoroscopic and echocardiographic guidance. In Patient 1, after stenting of the left middle lobar vein in crossover with the left superior pulmonary vein (provisional approach), residual ostial stenosis of the left superior vein required escalation to a culotte bifurcation strategy. Remaining lesions in both patients were treated with large‐diameter stents.

**Conclusions and Clinical Management:**

Early recognition using echocardiography and CT angiography is crucial. Endovascular interventions—particularly stent implantation—provide effective restoration of venous flow, but standardized protocols, long‐term patency assessment, and optimal antithrombotic therapy remain areas of ongoing investigation. Advances in coronary intervention may guide refined techniques for managing complex PVS. This paper presents a comprehensive clinical management approach, from symptom onset to definitive treatment.

## 1 Background

### 1.1 Introduction

Pulmonary vein stenosis or occlusion (PVS/O) is a rare but serious complication that can lead to significant morbidity and mortality if not promptly diagnosed and treated [1]. While historically associated with fibrosing mediastinitis or lupus, lymphoma, or leukemia [2], acquired PVS/O is now primarily linked to radiofrequency ablation (RFA) for atrial fibrillation (AF). Due to its rarity and frequent underdiagnosis, the true incidence is difficult to determine and varies from 1% to 21% according to reports [3, 4, 5]. Excessive thermal injury during RFA can trigger fibrosis, intimal proliferation, and subsequent venous stenosis or occlusion. PVS/O leads to increased pulmonary venous resistance and hemodynamic pulmonary hypertension, which can progress to pulmonary lobar edema and, in advanced stages, high mortality. While mild asymptomatic PVS may not require intervention, severe cases often necessitate transcatheter therapy, including balloon angioplasty (BA) and stenting. Stenting has demonstrated superior long‐term patency compared to BA, with lower rates of in‐stent restenosis [6]. However, no standardized treatment guidelines for PVS/O currently exist, and available literature on long‐term outcomes remains limited. Routine screening for PVS post‐RFA is not recommended by Heart Rhythm Society consensus statements [7]. Contrast‐enhanced CT is commonly used for diagnosis due to its high spatial resolution; however, CT alone may not reliably detect subtotal occlusions, as small residual microchannels may persist [8, 9].

This study aims to evaluate the presentation, diagnostic strategies, and treatment outcomes of PVS/O, with a particular focus on our single‐center experience. By analyzing our diagnostic approach, procedural success rates, and long‐term results following BA and stenting, we seek to contribute to the understanding and management of this complex condition.

### 1.2 Management of PVS/O

#### 1.2.1 Clinical Presentation

PVS/O presents with nonspecific symptoms that can be mistaken for common pulmonary conditions, leading to delays in diagnosis and treatment [10]. Symptoms typically develop a few months after the triggering event, such as RFA, with dyspnea, cough, fatigue, and decreased exercise tolerance being the most frequent manifestations. Chest pain and, less commonly, hemoptysis may also occur, particularly in cases complicated by venous congestion or infarction [11]. Imaging studies often reveal perfusion defects in the affected lung regions, with left‐sided pulmonary veins being more frequently involved. Due to the overlap with other pulmonary pathologies, misdiagnosis is common, with patients often initially treated for pneumonia, bronchitis, or even suspected malignancy, leading to unnecessary interventions such as antibiotic courses, bronchoscopy, or even thoracic biopsy [10, 12]. Early recognition of PVO is essential to prevent progression to total occlusion, which may limit therapeutic options.

#### 1.2.2 Diagnosis

The diagnosis of PVS/O requires a multimodal imaging approach to ensure accurate assessment and timely intervention. While computed tomography (CT) and magnetic resonance imaging (MRI) are commonly used for initial evaluation, CT alone may be insensitive to subtotal occlusions, as residual microchannels can still be present. Invasive pulmonary vein angiography plays a crucial role in further assessment, as it can identify treatable residual luminal patency in cases deemed occluded by CT [13, 14]. Additionally, transesophageal echocardiography (TEE) can be utilized for early screening, particularly in the presence of suggestive symptoms. When abnormal flow acceleration is detected on TEE, further imaging with CT or MRI is warranted to confirm and quantify the degree of stenosis. Early and precise diagnosis is essential, as timely intervention with stenting or BA can help maintain venous patency and reduce the risk of total occlusion and recurrence.

#### 1.2.3 PVS/O Treatment

The primary indication for pulmonary vein stenting is the presence of symptomatic PVS/O with a luminal narrowing exceeding 70%. This degree of stenosis leads to increased pulmonary venous pressure and impaired blood flow, often resulting in symptoms such as dyspnea, cough, hemoptysis, and reduced exercise tolerance [15]. Diagnosis is confirmed through invasive angiography, which provides a detailed assessment of stenosis severity, vessel anatomy, and the feasibility of intervention. Among available percutaneous treatments, stent placement is generally preferred over BA, particularly in cases of severe stenosis or subtotal occlusion. Stenting has demonstrated superior long‐term outcomes by reducing the risk of restenosis, maintaining vessel patency, and minimizing the need for repeat interventions [6]. Studies indicate that larger‐diameter stents offer the best results, significantly lowering the incidence of in‐stent restenosis compared to smaller stents. While procedural complications are comparable between BA and stenting, the latter provides greater durability and sustained symptom relief, making it the preferred treatment strategy [11, 14, 15]. Given the absence of standardized guidelines, current evidence supports stenting as the first‐line interventional therapy for severe PVS/O secondary to AF ablation. However, further studies are needed to refine patient selection criteria and optimize long‐term management strategies.

#### 1.2.4 Clinical Case Examples in Management

We present two cases of young individuals who, following radiofrequency catheter ablation for AF, experienced prolonged and challenging episodes of worsening dyspnea and productive cough, initially diagnosed as recurrent pneumonia but unresponsive to multiple courses of antibiotics.

### 1.3 Case 1

**Figure 1:**
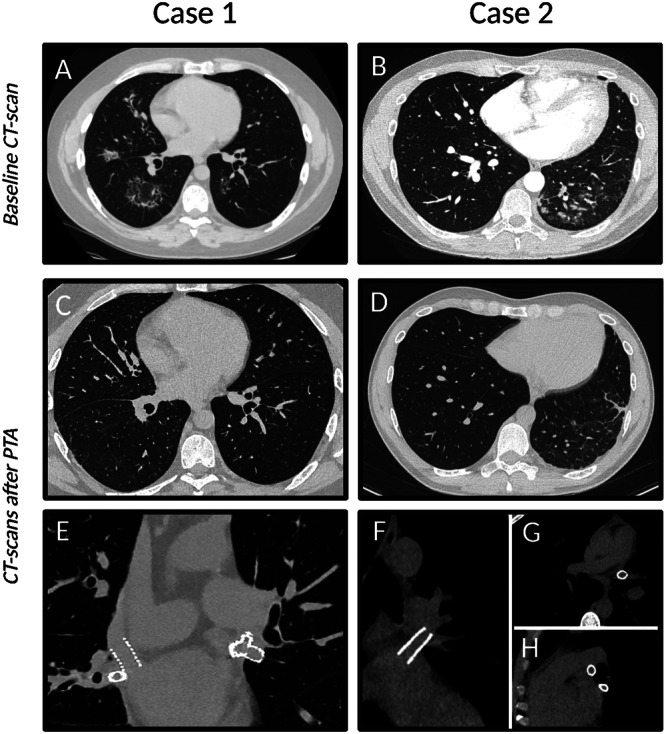
Role of CT in the diagnostic work‐up and follow‐up. In (A), the CT of Patient 1 shows a ground‐glass pattern throughout all the right lobes and involving the left inferior lobe. Also, Patient 2 repeated several CT scans, which constantly showed, as the main sign, a worsening obstructive pneumonia pattern in the left inferior lobe (B). At CT scan follow‐ups, we notice the complete resolution of the obstructive pneumonia pattern for patients 1 (C) and 2 (D). HRCT can offer high‐quality images and be essential to confirm the good results of Pulmonary veins PTA. HRCT of Patient 1 clearly shows the patency of both the stents in RSPV and RMLV, whose origin is separated, and the good result of the culotte technique used to treat the bifurcation of LSPV and LMLV (E). Similarly, we can appreciate the absence of restenosis of the stent in LSPV (F, H) and in the LIPV (G, H) for patient 2.

**Figure 2:**
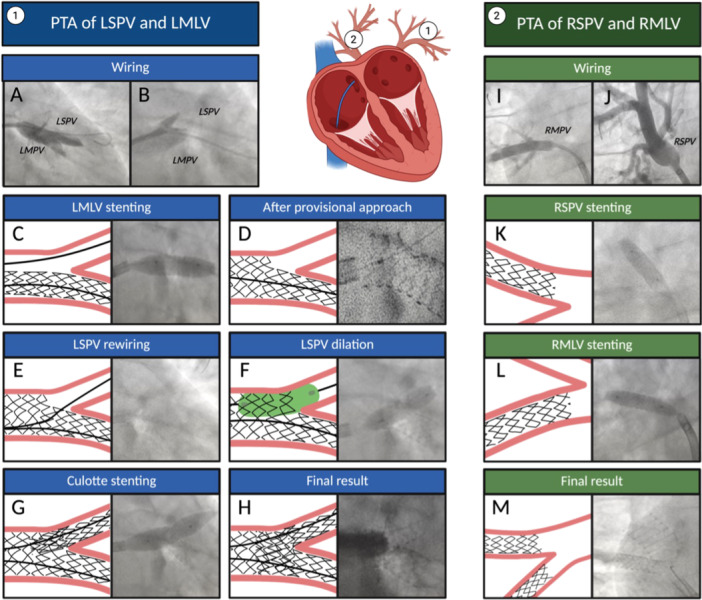
Percutaneous transluminal angioplasty (PTA) on LSPV‐LMLV (A–H) and RSPV and RMLV (I–M) in patient 1. After transseptal puncture, an angiographic guidewire 0.032″ was placed into LSPV (A) and a 0.018″ × 300 cm Connect guidewire into the LMLV (B). A Talon stent 8.0 × 17 mm was implanted into the LMLV, in crossover at the bifurcation with LSPV (C). We started with a provisional approach and, after having verified the correct opening of the stent struts (D), we rewired the LSPV with a BMW Universal 300 cm guidewire (E), and multiple dilations were performed (F). Due to residual stenosis at the ostium of the LSPV, we had to switch to a dual stent culotte technique by implanting a Talon QBX 10.0 × 17 mm stent (G). In (H), we can appreciate the two stents with correct strut opening across the bifurcation. Regarding PTA of the RSPV (J), a BMW Universal 300 cm guidewire was placed into the vessel and, after a predilation with a Trek 5.0 × 15 mm balloon, an unmedicated Talon QBX stent 5.0 × 17 mm was implanted (K). Similarly, an unmedicated Talon QBX stent 9.0 × 17 mm was implanted in the RMLV (I, L). In (M), the 2 stents are well visible in the right superior and middle lobar veins, which show separate origins. [Color figure can be viewed at wileyonlinelibrary.com]

A 37‐year‐old man with a history of paroxysmal AF underwent transcatheter ablation. One month after the electrophysiological procedure, he developed cough and dyspnea, initially attributed to chronic bronchitis with recurrent bilateral pneumonia. Therefore, he was treated with several courses of antibiotics. After his third episode of pneumonia, he was admitted to our Department of Respiratory Medicine for further diagnostic evaluation. Physical examination revealed coarse crackles, while a chest X‐ray showed diffuse consolidations and pleural effusion. Spirometry tests were largely normal, with only a mild reduction in carbon monoxide (CO) transfer. Echocardiography showed signs of pulmonary hypertension, such as flattening of the interventricular septum, a systolic pulmonary arterial pressure of 35 mmHg, and a pulmonary acceleration time of 60 ms. Multi‐slice spiral chest CT (MSCT) revealed heterogeneous consolidation with scattered ground‐glass opacities in the left superior lobe (anterior segment) and left inferior lobe (posterior segment), as well as a ground‐glass pattern throughout all the right lobes (Figure 1A). Bilateral apical pleural effusion was also present. Additionally, at CT angiography there were opacification delays in the pulmonary veins, suggesting severe stenosis of the right middle lobar vein (RMLV, an accessory branch distinct from the right superior pulmonary vein [RSPV]) and the RSPV, mild stenosis of the right inferior pulmonary vein (RIPV), and severe stenosis of the left superior pulmonary vein (LSPV). After referring to our Heart Team, the patient was treated with percutaneous transluminal angioplasty. The entire procedure was guided by TEE visualization. After transseptal puncture, a steerable sheath 8.5 Fr catheter was placed in the left atrium, and a 0.018″ × 300 cm Connect guidewire was advanced into the left middle lobar vein (LMLV, an accessory branch distinct from the LSPV) (Figure 2A,B). A Talon stent 8.0 × 17 mm (9 atm) was implanted into LMLV, in crossover at the bifurcation with the LSPV (Figure 2C). Due to evidence of severe residual stenosis at the ostium of LSPV with flow acceleration on TEE (Figure 2D), the same was wired with a BMW Universal 300 cm guidewire (Figure 2E); multiple dilations of LSPV were performed (Figure 2F). Due to residual stenosis, an unmedicated Talon QBX 10.0 × 17 mm stent (9 atm) was implanted using the “culotte” technique (Figure 2G,H). For the treatment of RSPV and RMLV (which had a separate origin from the left atrium) stenoses (Figure 2I,J), after a predilation with a semi‐compliant 5.0 × 15 mm balloon (12 atm) an unmedicated peripheral stent 5.0 × 17 mm (9 atm) was then implanted into RSPV (Figure 2K). Thereafter, an unmedicated peripheral stent 9.0 × 17 mm (11 atm) was implanted (Figure 2L) into RMLV, and post‐dilation was achieved with an Admiral Extreme 12.0 × 20 mm balloon (8 atm). The final result showed no residual stenosis (Figure 2M).

The patient was discharged with dual antiplatelet therapy with aspirin and clopidogrel for at least 6 months. He had his first follow‐up at 4 months (December 2023) and a second follow‐up at 8 months (April 2024). In both visits, he reported being finally relieved of all respiratory symptoms and having a good quality of life. He underwent a high‐resolution CT scan, which clearly showed the resolution of the ground‐glass pattern (Figure 1C) and the patency of both the stents placed into RSPV and RMLV, and the good results of the culotte technique performed to treat the bifurcation between LSPV and LMLV (Figure 1E). Transthoracic echocardiography (TTE) showed good parameters, with peak velocities of pulmonary vein flows less than 1 m/s.

### 1.4 Case 2

**Figure 3:**
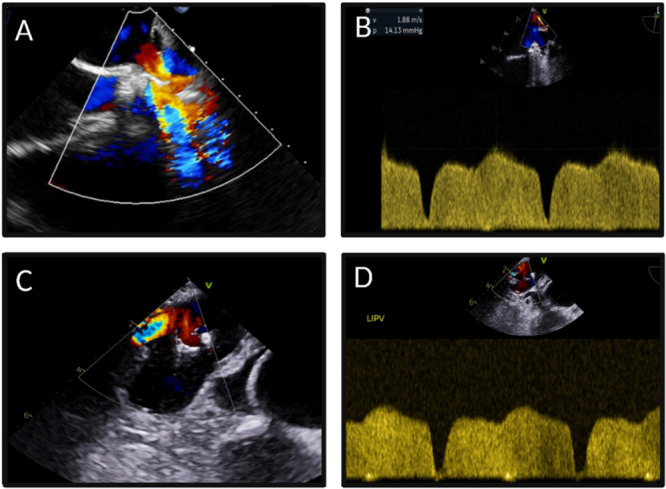
Transesophageal echocardiogram (TEE) in the diagnostic work‐up of patient 2. TEE revealed an important flow acceleration mainly in the LSPV with aliasing at color Doppler (A), and a peak velocity of 2 m/s, a peak gradient of 14 mmHg (B), suggesting pulmonary vein stenosis. Similarly, flows were accelerated in the LIPV (C), with a velocity of 1.6 m/s (D). [Color figure can be viewed at wileyonlinelibrary.com]

The second case concerns a 34‐year‐old man who had experienced recurrent pneumonia since 2021, leading to a thoracoscopic, CT‐guided biopsy revealing diffuse septal thickening and pulmonary fibrosis with the typical pattern of cryptogenic organizing pneumonia (COP). He was treated with steroids and azathioprine, but without any real clinical improvement. Quantiferon and autoimmune tests were negative. Repeated chest CT scans showed a consolidation in the left inferior lobe (Figure 1B). He was admitted to our hospital in April 2024. Physical examination revealed nonspecific, harsh breath sounds. Upon reviewing his history, it was discovered that he had undergone redo catheter ablation for AF in 2021, about 1 month before the onset of respiratory symptoms. The diagnostic workup included an entry chest X‐ray, which confirmed consolidation in the left inferior lobe and bilateral pleural effusion, and TEE, which revealed increased flow velocities in the pulmonary veins, most notably in the LSPV, with a peak velocity of 2 m/s, a peak gradient of 14 mmHg (Figure 3A,B) and in the left inferior pulmonary vein (LIPV), with a peak velocity of 1.6 m/s (Figure 3C,D). All these findings were highly suggestive of pulmonary vein stenosis (PVS).

**Figure 4:**
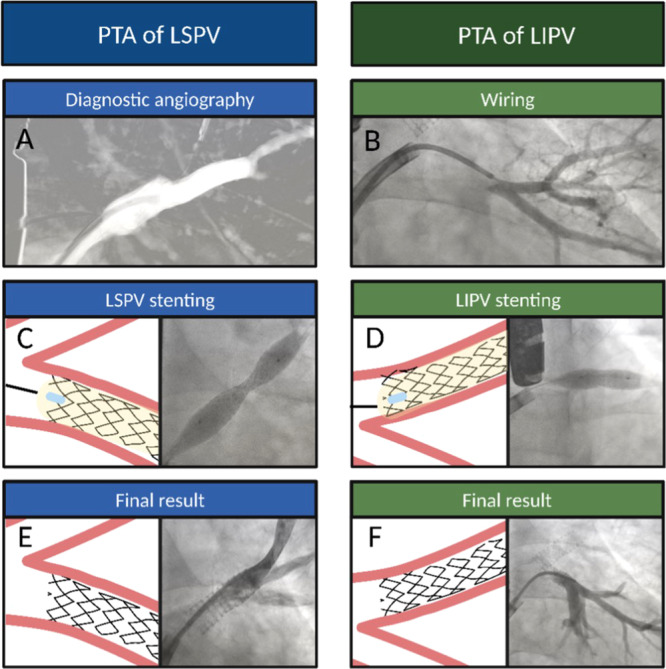
Percutaneous transluminal angioplasty (PTA) of LSPV and LIPV in patient 2. At angiography (A), LSPV stenosis was clearer, so, after predilation, a QBX 10.0 × 26 mm stent was implanted (C, E) in the ostial‐proximal segment. For LIPV, the key passages were diagnostic angiography (B), predilation with the same Admiral 6.0 × 40 mm balloon, and QBX 8.0 × 17 mm stent implantation (D, F). [Color figure can be viewed at wileyonlinelibrary.com]

As in the first case, the Heart Team gave an indication for percutaneous transluminal angioplasty, using TEE visualization to guide the procedure. After a transseptal puncture, a steerable sheath 8.5 Fr was placed in the left atrium. We began by treating LSPV (Figure 4A): after predilation of the lesion with a semi‐compliant 6.0 × 40 mm balloon (12 atm), a peripheral stent 10.0 × 26 mm (9 atm) was implanted in the ostial‐proximal segment (Figure 4B,C). Then we focused on LIPV, again protecting the middle lobar one through the 0.018″ × 300 cm Connect guidewire (Figure 4D). Predilation was achieved with a semi‐compliant 6.0 × 40 mm balloon (12 atm). Finally, an unmedicated peripheral stent 8.0 × 17 mm (9 atm) was implanted in the ostial‐proximal tract (Figure 4E,F).

This patient was also discharged on dual antiplatelet therapy (aspirin plus clopidogrel) for at least 6 months. At a follow‐up visit at 2 months after the procedure, he showed general clinical improvement, with the disappearance of the cough but persistence of exertional dyspnea (NYHA II), palpitations, and frequent supraventricular extrasystoles on ECG Holter. After the introduction of an antiarrhythmic drug (flecainide), he experienced consistent improvement, and at his subsequent follow‐up, he was asymptomatic for both palpitations and dyspnea. High‐resolution chest CT showed complete resolution of the obstructive pneumonia pattern in the left inferior lobe (Figure 1D). Moreover, it clearly demonstrated the absence of any restenosis of the stents in the left superior and inferior veins, with particularly good quality scans (Figure 1F–H). TTE was normal, with no signs of pulmonary vein flow acceleration.

## 2 Discussion

PVS and pulmonary vein occlusion (PVO) are complications following AF catheter ablation, caused by thermal injury to tissues surrounding the pulmonary veins and subsequent scar tissue formation. The extent and location of these scars determine the severity of PV narrowing. According to a previous study [1], the median time from AF catheter ablation to the development of these complications is 7.5 weeks, though in our two patients, it occurred after 1 month.

The diagnosis of PVS or PVO is often missed or delayed, and early recognition relies primarily on clinical awareness. The severity of symptoms does not necessarily correlate with the anatomical degree of stenosis but rather with the overall vascular involvement. For instance, a single‐vessel stenosis may cause mild or absent symptoms, as it can be compensated by the remaining pulmonary circulation.

Various imaging techniques can assist clinicians; however, an accurate diagnosis typically requires a comprehensive assessment of the patient's clinical history, risk factors, and the integration of multiple diagnostic tests [2].

Common findings on chest X‐rays include consolidation shadows and pleural effusions, which are not specific to PVS and are often attributed to pulmonary infections [3, 4]. TTE may reveal pulmonary hypertension and right‐heart failure, as the backflow obstruction in the PVs increases pulmonary capillary pressure. TEE can be more sensitive in detecting increased peak Doppler flow velocity, particularly in the left superior PV, as observed in our second patient. TEE is also valuable for follow‐up assessments to detect in‐stent or in‐vein restenosis [5]. However, multi‐slice CT (MSCT) tends to offer better sensitivity and can help identify the distribution of PVs. The advantages of MSCT over MRI include lower cost and quicker image acquisition, although MRI may be preferable for long‐term follow‐up [16]. Ventilation‐perfusion (V/Q) scans can provide useful insights into lung function, revealing perfusion defects in cases of severe stenosis. However, V/Q scans may be inconclusive in cases of bilateral PVS, as the percentage of flow to each segment is dependent on flow to the other segments [17].

Ultimately, while CT pulmonary angiography and V/Q scans provide valuable diagnostic information, they should not be solely relied upon, as clinical judgment remains essential for accurate diagnosis and decision‐making. Pulmonary catheter angiography is often necessary to confirm the diagnosis. In our second case, the lack of awareness of PVS led to thoracoscopic surgery and biopsy, which revealed interstitial fibrosis, septal thickening, accumulation of hemosiderin‐laden macrophages in the alveoli, and histological changes in the intima and media of pulmonary veins and arteries—all secondary to pulmonary venous outflow impairment.

While diagnosing PVS is the first challenge, managing it poses even greater difficulties. The consensus is that symptomatic, severe PVS should be treated with angioplasty. However, the management of severe asymptomatic stenoses is more controversial, although many studies suggest clinical benefits. The recommended approach for asymptomatic PVS is regular monitoring (via imaging) every 3–6 months, with treatment considered once stenosis becomes severe [18].

Previous studies have shown the safety and effectiveness of BA, though the rate of restenosis remains high (47%–61%) [1, 2]. Similar to coronary artery disease, the use of stents in PVS appears to reduce restenosis rates (ranging from 0% to 47%) [6, 19, 20], with better outcomes reported when stents larger than 9 mm in diameter are used [6, 19, 20]. In our second case, we performed standard angioplasty with balloon predilation, followed by stent placement and post‐dilation. The first case, however, required a more complex “bifurcation technique,” where a provisional approach [21] (with a protective guidewire on the LSPV) was converted to a double‐stent approach using the “culotte” technique. This is an off‐label procedure, as evidence in this area is still lacking. Nevertheless, this case demonstrates the potential application of techniques proven successful in the coronary field to this rarer and more complex setting.

Future research should focus on confirming the long‐term benefits of percutaneous coronary intervention (PCI) with stenting in PVS, providing clearer guidelines for when to perform pulmonary angioplasty, and expanding our knowledge of specific techniques for various scenarios (e.g., bifurcations, trifurcations, complex lesions, and restenosis), like the advancements made in coronary interventions.

In this context, it is important to acknowledge the contextual scope of our findings. Although our observations derive from a single‐center experience and are limited to two illustrative cases, they highlight recurring diagnostic and procedural patterns that may be transferable to similar tertiary referral settings. The rarity of post‐ablation PVS inherently restricts broader generalizability; nonetheless, the structured diagnostic workflow and interventional strategy described here can serve as a practical reference for multidisciplinary “Heart Team” management.

Beyond the two cases presented, our center has evaluated additional patients with suspected post‐ablation PVS over the past 5 years. Among these, the diagnosis was confirmed in approximately one‐third, and most underwent multimodal imaging, including TEE and CT angiography. Endovascular treatment was performed in selected symptomatic patients, achieving procedural success in all cases without periprocedural complications. During follow‐up, no deaths or early restenosis were observed, and all treated patients reported sustained symptomatic improvement. Although these observations are preliminary and descriptive, they support the feasibility and reproducibility of our diagnostic and interventional workflow. Ultimately, multicenter registries and prospective collaborations are needed to better quantify procedural success, restenosis rates, and long‐term outcomes under standardized follow‐up protocols.

**Figure 5:**
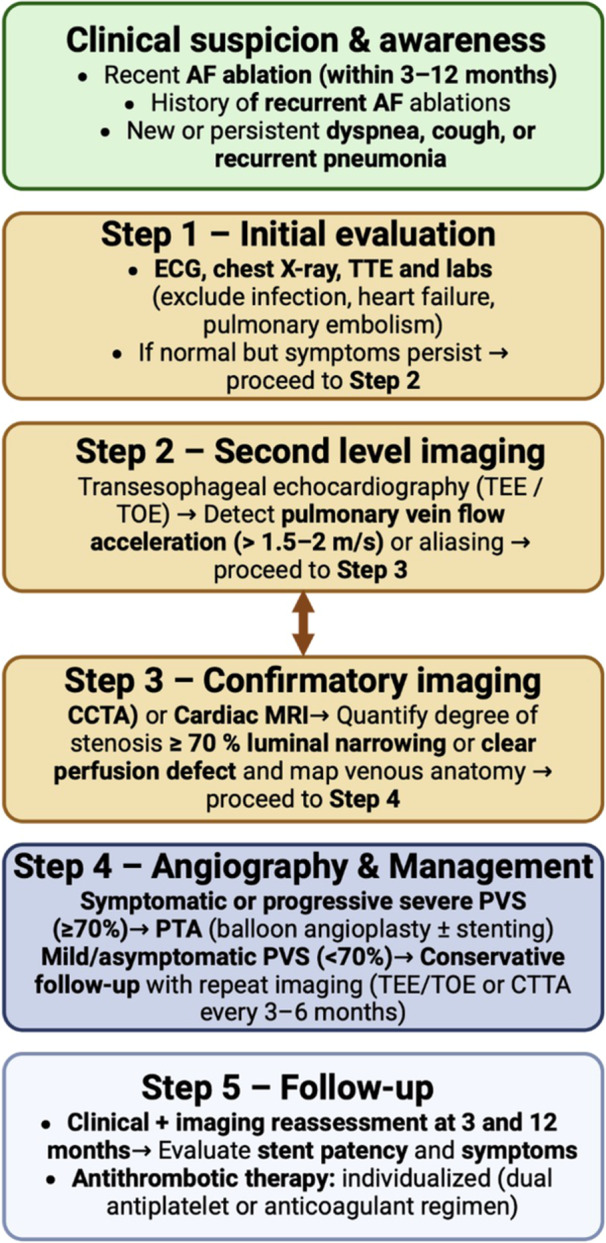
Proposed diagnostic and management algorithm for post‐ablation pulmonary vein stenosis (PVS). The flow chart outlines a structured stepwise approach from clinical suspicion to definitive management within a multidisciplinary Heart Team framework. TEE/TOE and CT angiography are central for diagnosis, while percutaneous angioplasty is reserved for severe or symptomatic stenoses. [Color figure can be viewed at wileyonlinelibrary.com]

Based on our institutional experience, we propose a pragmatic “Heart Team” diagnostic and management algorithm (Figure 5) for post‐ablation PVS, designed to support timely recognition and standardized care.

The process begins with clinical suspicion, typically triggered by new or persistent dyspnoea, cough, or recurrent pneumonia occurring within 3–12 months after AF ablation, particularly in patients with a history of multiple procedures.

Step 1 – Initial evaluation:

A basic assessment with ECG, chest X‐ray, and laboratory testing is performed to exclude common pulmonary or infectious causes such as pneumonia, heart failure, or pulmonary embolism.

If findings are unremarkable but symptoms persist, proceed to Step 2.

Step 2 – Second level imaging:

Transoesophageal echocardiography (TEE/TOE) is used to evaluate pulmonary venous flow and detect flow acceleration (> 1.5–2 m/s) or aliasing suggestive of hemodynamically significant stenosis.

If abnormal findings or persistent clinical suspicion are present, proceed to Step 3.

Step 3 – Confirmatory imaging:

Contrast‐enhanced CT angiography (CCTA) or cardiac MRI is performed to quantify the degree of luminal narrowing and to map pulmonary venous anatomy.

Cases with ≥ 70% stenosis or clear perfusion defects should advance to Step 4.

Step 4 – Contrast‐enhanced CT angiography Heart Team decision and management:

All eligible cases are discussed within a multidisciplinary Heart Team (interventional cardiology, radiology, pulmonology, and electrophysiology). Pulmonary angiography enables definitive diagnosis and, when indicated, treatment during the same procedure.

- Symptomatic or progressive severe PVS (≥ 70%) → Percutaneous treatment with BA ± stenting.
- Mild or asymptomatic PVS (< 70%) → Conservative follow‐up with repeat imaging (TEE/TOE or CCTA) every 3–6 months.

Step 5 – Follow‐up:

Patients undergo clinical and imaging reassessment at 3 and 12 months to evaluate stent patency, restenosis, and symptom improvement.

Antithrombotic therapy (dual antiplatelet or anticoagulant regimen) is individualized according to patient profile and procedural characteristics.

This structured pathway enables timely recognition, multidisciplinary consensus, and standardized follow‐up, helping to reduce delays and optimize outcomes in this rare but challenging condition.

Another important consideration is antithrombotic management. Notably, our first patient discontinued anticoagulant therapy (rivaroxaban) exactly 1 month prior to their clinical deterioration. A review of the literature suggests that delayed or missed diagnoses of PVS often lead to the premature discontinuation of oral anticoagulation, which may worsen the condition [13]. Therefore, careful attention should be paid to prophylactic anticoagulation following transcatheter AF ablation, with discontinuation only considered if imaging confirms the absence of even asymptomatic PVS.

While ample evidence supports well‐defined guidelines for antiplatelet therapy following coronary stenting, fewer studies focus on therapies after endovascular stenting for PVS or PVO. Some studies recommend anticoagulants due to concerns about venous thrombosis, while others suggest dual antiplatelet therapy, arguing that stents placed in low‐flow systems may benefit from this approach, and data on venous drug‐eluting stent (DES) thrombosis is limited. A uniform consensus on the optimal duration of therapy is still lacking. Anticoagulation proponents suggest at least 1 year of treatment with warfarin [22, 23], with lifelong therapy for stents smaller than 1 cm [23]. Those favoring dual antiplatelet therapy recommend at least 6 months, with reevaluation based on follow‐up imaging (CT and echocardiography) and the patient's overall thrombotic and ischemic risk [24, 25]. Future research is crucial to clarify these important issues.

## 3 Conclusions

In conclusion, both patients experienced significant improvement in symptoms and pulmonary function after stent implantation. However, the management of PVS following AF ablation remains a complex and evolving field requiring further research, particularly in long‐term outcomes and the best strategies for antithrombotic management. Moreover, the extension of angioplasty to PVS treatment represents a promising frontier, opening new avenues for care and expanding the indications for interventional approaches in this challenging condition.

## Funding

The authors received no specific funding for this work.

## Consent

Written informed consent was obtained from the patients.

## Conflicts of Interest

Francesco Burzotta discloses having received speaker's fees from Abbott, Abiomed, Medtronic, Edwards Lifesciences, and Johnson & Johnson. Cristina Aurigemma discloses having received speaker's fees from Abbott, Abiomed, Medtronic, Edwards Lifesciences, Johnson & Johnson, and Daiichi Sankyo. Carlo Trani discloses having received speaker's fees from Abbott, Abiomed, Johnson & Johnson Medtronic, Boston Scientific, and Daiichi Sankyo. The other authors declare no conflicts of interest.
